# Beyond Gastroenteritis: Successful Management of a Salmonella-Induced Mycotic Aneurysm

**DOI:** 10.7759/cureus.102375

**Published:** 2026-01-27

**Authors:** Tinatin Jomidava, Lasha Mukhigulashvili, Giorgi Kentchadze, Lasha Okujava, Rati Kurdadze, Giorgi Japharidze, Davit Arsenidze

**Affiliations:** 1 Infectious Disease, Georgian National University, Tbilisi, GEO; 2 Pharmacology, European University, Tbilisi, GEO; 3 Infectious Disease, Caucasus Medical Centre, Tbilisi, GEO; 4 Vascular Surgery, American Hospital Tbilisi, Tbilisi, GEO; 5 Cardiac Surgery, Chapidze Cardiac Center, Tbilisi, GEO

**Keywords:** aaa rupture, abdominal aortic aneurysms, mycotic aortic aneurysm, salmonella aortitis, salmonella infection

## Abstract

*Salmonella *species are an uncommon cause of mycotic aneurysms and invasive sepsis, with human infection most often acquired through ingestion, and parenteral transmission being exceedingly rare. We describe a middle-aged farmer who developed *Salmonella *bacteremia and an infected infrarenal abdominal aortic aneurysm following an accidental needle-stick injury while vaccinating cattle. Blood cultures and intraoperative tissue specimens yielded *Salmonella *species. Despite appropriate targeted antimicrobial therapy, the disease progressed rapidly, resulting in rupture of the abdominal aorta that required emergent aortic ligation and axillo-bifemoral bypass, complicated by bowel ischemia and necrosis necessitating left hemicolectomy, colostomy, subsequent re-resection, and acute kidney failure requiring hemodialysis. After multiple surgical interventions, prolonged intensive care, and extended antimicrobial treatment, the patient eventually stabilized and was discharged in satisfactory condition, underscoring the aggressive nature of *Salmonella*-associated vascular infections and the need to consider rare zoonotic and occupational transmission routes in agricultural workers.

## Introduction

*Salmonella *species are Gram-negative enteric pathogens that most commonly cause self-limited gastroenteritis and, less frequently, invasive infections such as bacteremia, endovascular infection, and focal metastatic disease [1]. Mycotic aneurysms caused by *Salmonella *spp. are rare but well-documented, accounting for a small proportion of infected aneurysms, with a predilection for the abdominal aorta [2]. These infections are associated with high morbidity and mortality due to rapid aneurysmal expansion, rupture, and the need for complex surgical and antimicrobial management [3].

Human infection with *Salmonella *typically occurs via the fecal-oral route following ingestion of contaminated food or water [4]. Zoonotic transmission is well recognized, particularly in individuals with occupational exposure to livestock; however, transmission through direct parenteral inoculation is exceedingly uncommon and sparsely reported in the literature [1,2]. Accidental needle-stick injuries in agricultural settings represent a potential but underappreciated mechanism for introducing pathogenic organisms directly into the bloodstream, bypassing gastrointestinal defenses and potentially leading to fulminant systemic infection.

Invasive *Salmonella *infectionis a known risk factor for endovascular seeding, especially in the presence of preexisting vascular abnormalities such as atherosclerosis or aneurysmal disease. Once established, Salmonella-associated mycotic aneurysms tend to follow an aggressive clinical course, often complicated by sepsis, aneurysmal rupture, and adjacent organ ischemia. Early diagnosis can be challenging, and management typically requires prolonged targeted antimicrobial therapy combined with urgent surgical intervention [2].

Here, we report a rare and severe case of *Salmonella *bacteremia and mycotic abdominal aortic aneurysm following accidental parenteral inoculation via a needle-stick injury sustained during cattle vaccination. The case highlights an unusual route of zoonotic transmission and illustrates the devastating vascular and gastrointestinal complications that can result. This report aims to raise awareness of this rare mechanism of infection and to emphasize the importance of occupational safety and early recognition of invasive zoonotic infections in high-risk populations such as agricultural workers.

## Case presentation

The patient presented to the clinic with a two-week history of abdominal and back pain. Physical examination revealed abdominal distension and tenderness, most pronounced in the right upper quadrant.

Two weeks prior to admission, the patient had undergone computed tomography (CT), which demonstrated an abdominal aortic aneurysm (AAA) measuring up to 3 cm. The patient was evaluated by a vascular surgeon, and urgent surgical intervention was not recommended at that time.

**Figure 1:**
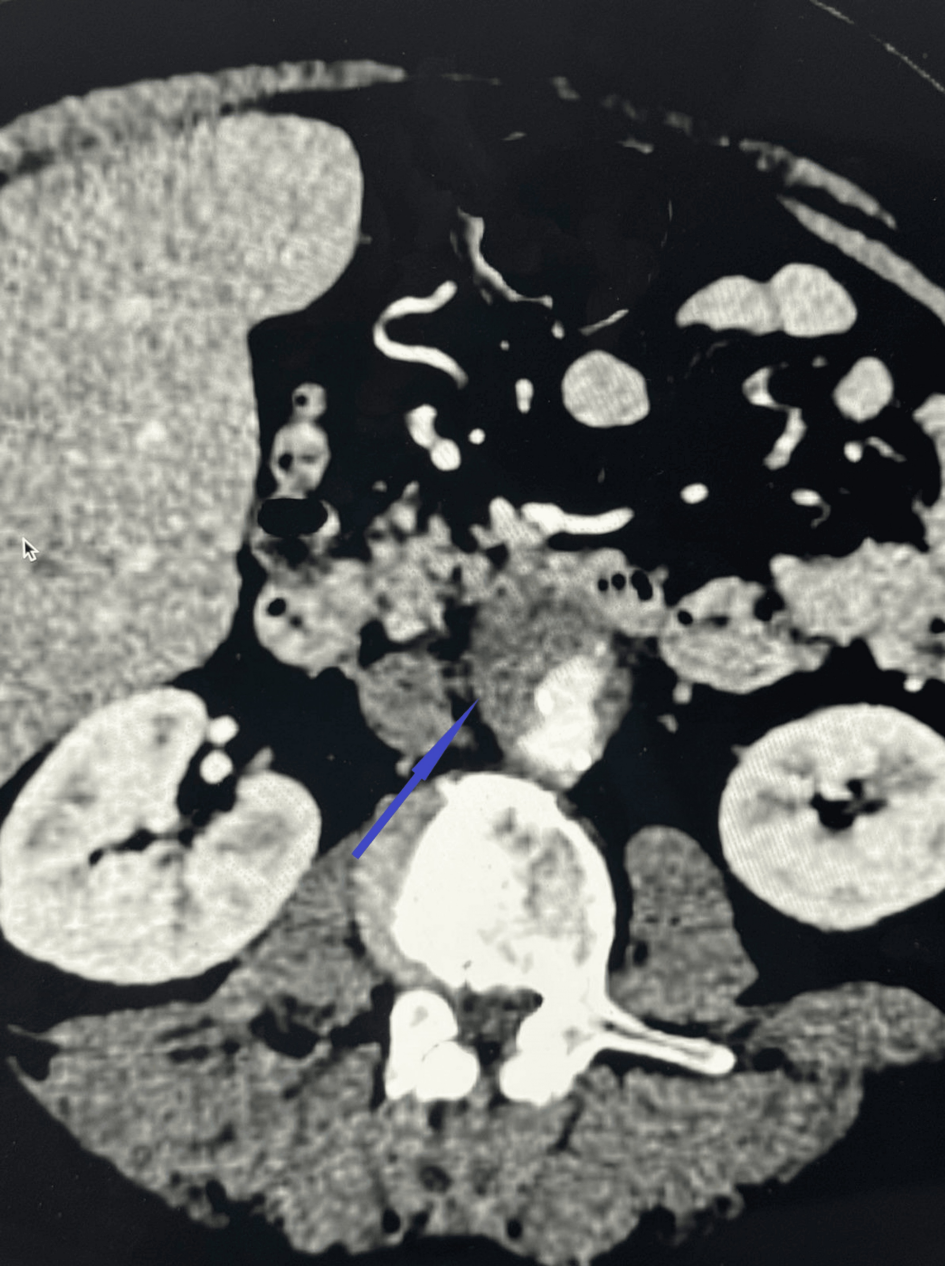
Abdominal computed tomography angiography (CTA) Abdominal computed tomography angiography (CTA) demonstrating changes in the distal abdominal aorta most consistent with a penetrating atherosclerotic ulcer (blue arrow). These findings are accompanied by infiltration of the perivascular adipose tissue, extending paraaortically and involving the level of the bilateral common iliac arteries.

Given the severity of pain and the patient’s medical history, contrast-enhanced CT angiography (CTA) of the abdomen was performed. Imaging revealed pathological changes in the distal abdominal aorta, which were most consistent with a penetrating atherosclerotic ulcer (Figure 1). The patient was again assessed by a vascular surgeon, and urgent intervention was not indicated at that stage.

However, due to the presence of severe sepsis, antimicrobial therapy was initiated. The patient was started on ceftriaxone 2 g intravenously every 24 hours and vancomycin 1 g intravenously every 12 hours. Two sets of blood cultures were obtained, and both samples grew non-typhoidal *Salmonella *spp. Additionally, cardiac ultrasound did not reveal any notable changes. Consequently, vancomycin was discontinued. 

Over the subsequent days, the patient’s clinical condition progressively deteriorated, accompanied by worsening laboratory parameters, including marked leukocytosis with neutrophilia and a significant decline in hemoglobin and erythrocyte levels (Table 1). In response to this deterioration, a repeat contrast-enhanced CT scan of the abdomen was performed. Repeated blood cultures were performed. Antimicrobial therapy was modified to piperacillin-tazobactam.

**Table 1:** Laboratory test results during hospitalisation

Test	Unit	Normal Range	Day 1	Day 4	Day 7	Day 8	Day 10	Day 11	Day 17	Day 18	Day 37
WBC	10^9/L	4.00 - 9.00	10.23	11.57	16.77	8.73	12.34	6.52	20.34	19.48	12.18
Neutrophils	10^9/L	2.00 - 6.80	9.50	9.30	12.39	7.04	10.86	4.97	16.99	16.43	7.20
RBC	10^12/L	4.40 - 5.80	4.89	4.72	3.52	3.17	2.46	2.52	2.74	2.65	2.80
Hemoglobin	g/dL	13.50 - 18.00	15.0	14.6	10.9	9.3	7.2	7.4	8.0	7.8	8.2
C-reactive protein	mg/L	<5	150.48	147.22		258.18		306.01	274.44	259.82	136.89
Procalcitonin	ng/mL	<5	11.46	1.99				10.44	4.40	3.02	0.437
Lactate	Mmol/L	0.9 - 1.7	1.0		3.4	4.4	1.2	1.9	1.4	1.4	1.2
pH		7.31 - 7.43	7.42		7.27	7.43	7.34	7.39	7.46	7.36	7.44

**Figure 2:**
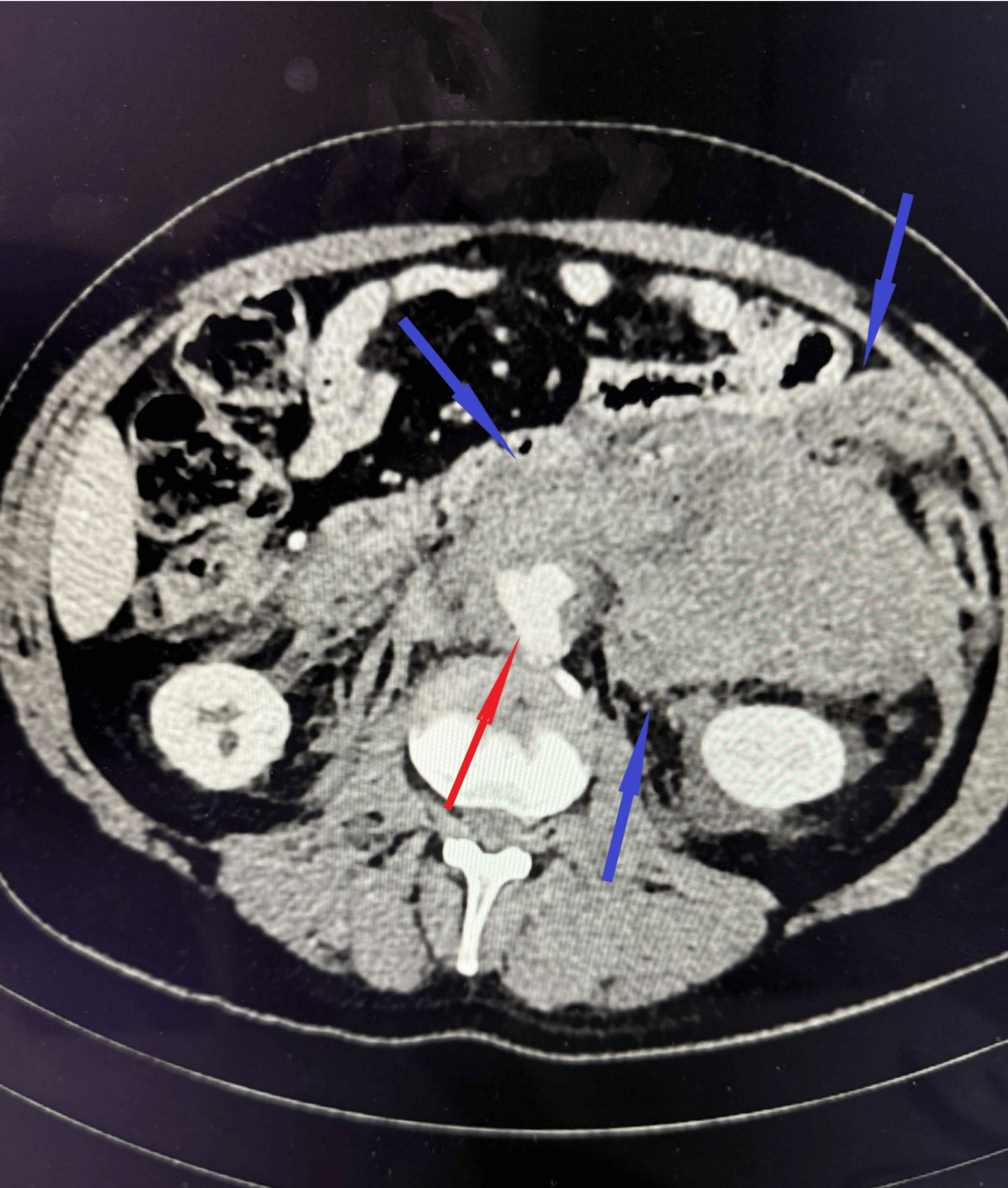
Contrast-enhanced CT Contrast-enhanced CT demonstrating a distal abdominal aortic aneurysm (red arrow) at the level of the aortic bifurcation, measuring approximately 40 × 40 mm with a length of ~35 mm, irregular contours, and an associated mural thrombus (7-8 mm). Extensive bilateral para-aortic retroperitoneal hemorrhage, more pronounced on the left, is present, forming a large organized hematoma (blue arrows) measuring 8.7 × 15.2 × 25.3 cm.

Imaging demonstrated a 40 × 40 mm aneurysm of the distal abdominal aorta at the level of bifurcation, extending approximately 35 mm in length. A large volume of paraaortic hemorrhage was identified, extending bilaterally into the retroperitoneal space, more pronounced on the left, where a partially organized retroperitoneal hematoma was observed. No active contrast extravasation was detected (Figure 2). Additionally, imaging revealed dissection of the right common iliac artery, with thrombosis of the false lumen.

The patient was taken emergently to the operating room. Surgical management included ligation of the infrarenal abdominal aorta and both common iliac arteries, followed by axillo-bifemoral bypass grafting. During surgery, the aneurysm sac was opened, and samples were obtained for bacteriological culture. Hemorrhage was noted in the retroperitoneal space; the area was packed with two surgical tampons, a laparostomy was performed, and the wound was covered with a sterile dressing.

Two days later, the laparostomy was reopened, and the retroperitoneal hematoma was evacuated. Intraoperative exploration revealed ischemia and necrosis of the sigmoid colon wall. Consequently, resection of the descending and sigmoid colon was performed. The distal stump was ligated and closed, and a transverse colostomy was created through a separate incision on the right lateral abdominal wall.

**Figure 3:**
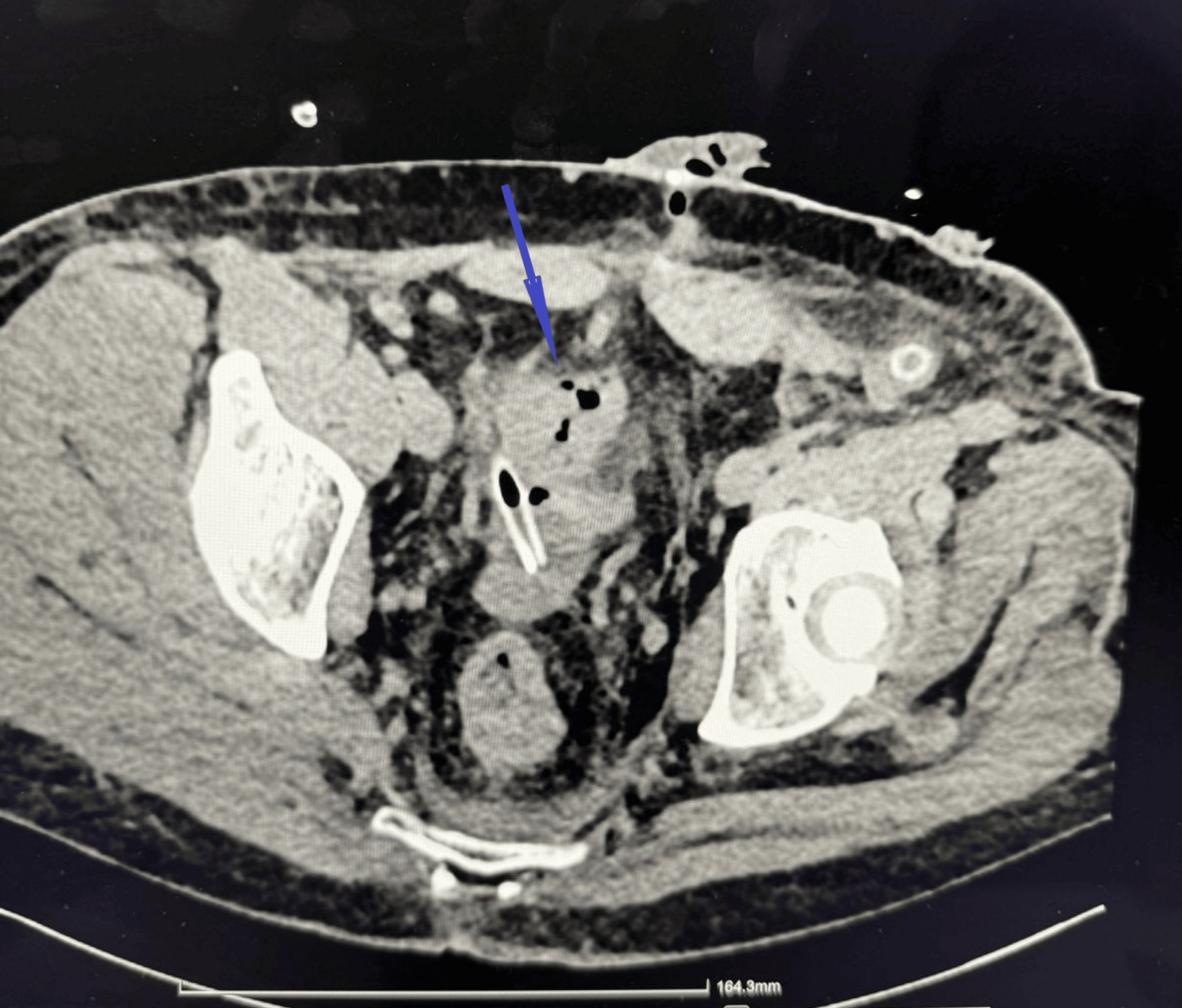
Abdominal CT Abdominal CT demonstrating a wall defect at the left sigmoid colon stump (blue arrow), measuring up to 4 mm in transverse diameter, with an adjacent isolated gas-density focus. A small volume of free fluid is present in the pelvic cavity near the superior margin of the urinary bladder, with associated gas-density foci, concerning for an anastomotic leak.

Four days later, a contrast-enhanced CT scan of the abdomen demonstrated edematous and mildly thickened walls of the sigmoid stump (Figure 3). A wall defect measuring up to 4 mm was identified on the left side of the stump, with an adjacent small gas collection. Additionally, a small amount of free intraperitoneal fluid and gas was observed in the pelvic cavity. These findings were suggestive of stump insufficiency (leak). The patient’s condition deteriorated acutely, and he was taken to the operating room with a diagnosis of acute peritonitis. Surgical revision revealed necrotic areas of the rectal ampullary stump, with disruption of bowel wall integrity. Resection within viable tissue margins was performed. Postoperatively, piperacillin-tazobactam therapy was initiated.

Microbiological analysis of the wound swab revealed extended-spectrum beta-lactamase (ESBL)-producing *Escherichia coli*. Accordingly, antimicrobial therapy was modified: piperacillin-tazobactam was discontinued, and meropenem was initiated.

After 20 days, the patient’s clinical condition and laboratory parameters returned to normal. The second set of blood cultures was negative. The patient was discharged in stable condition under the outpatient follow-up of a vascular surgeon and an infectious disease physician. A minimum of 4 weeks of antibiotics was recommended with the possibility of an oral step-down regimen.

## Discussion

*Salmonella-*associated mycotic aneurysms represent a rare but highly aggressive form of endovascular infection, characterized by rapid progression, high rates of rupture, and substantial mortality [5,6,7]. While *Salmonella *species are most commonly implicated in self-limited gastrointestinal disease, invasive infections can occur, particularly in the presence of bacteremia and underlying vascular pathology [8,9]. 

The present case is remarkable not only for the severity of vascular and gastrointestinal complications but also for the exceptionally unusual route of transmission-direct parenteral inoculation via an accidental needle-stick injury during cattle vaccination. Unusual route of transmission and pathogenesis of human salmonellosis overwhelmingly results from fecal-oral transmission [9,10]. Zoonotic transmission from livestock is well recognized but typically occurs through ingestion of contaminated animal products. In contrast, parenteral transmission of *Salmonella *is exceedingly rare, with only sporadic cases reported following laboratory accidents, contaminated injections, or penetrating trauma [11].

In this case, direct intravascular or deep tissue inoculation likely bypassed gastrointestinal immune barriers, enabling immediate systemic dissemination and bacteremia. This mechanism may explain the fulminant course observed. Parenteral inoculation allows for a high bacterial load to enter the bloodstream, facilitating rapid endothelial adhesion and vascular seeding. *Salmonella *spp. possess virulence factors, such as fimbriae, outer membrane proteins, and the ability to survive intracellularly within macrophages, which promote persistence in the vascular wall and predilection for atherosclerotic or aneurysmal segments of the aorta [5,7].

Invasive *Salmonella *infection is a well-established cause of mycotic aneurysms, particularly in older patients and those with pre-existing vascular disease [5,6]. Even small or previously stable aneurysms may rapidly enlarge or rupture following microbial seeding [6,7]. In the present case, the patient’s initially modest (3 cm) abdominal aortic aneurysm progressed to a ruptured, infected aneurysm within a short time frame, underscoring the destructive nature of *Salmonella*-induced arteritis. The inflammatory cascade triggered by infection of the arterial wall leads to medial necrosis, weakening of the vessel structure, and rapid aneurysmal expansion [1]. Imaging findings initially suggestive of a penetrating atherosclerotic ulcer may have represented early infectious involvement, highlighting the diagnostic challenge of distinguishing sterile vascular pathology from early mycotic disease [7].

Early diagnosis of *Salmonella *mycotic aneurysm is often difficult due to nonspecific symptoms such as abdominal or back pain, fever, and sepsis [6]. Blood cultures play a critical role and are positive in the majority of cases. In this patient, persistent *Salmonella *bacteremia despite appropriate antimicrobial therapy should have raised early concern for an endovascular focus [6,7]. Serial imaging is essential, as initial CT findings may be subtle. Rapid aneurysmal enlargement, periaortic inflammation, retroperitoneal hematoma, and vessel wall irregularity are key radiologic features [7]. This case illustrates the importance of maintaining a high index of suspicion for an infected aneurysm in any patient with *Salmonella *bacteremia and compatible symptoms, even when the aneurysm size is below conventional thresholds for surgical intervention.

Optimal management of *Salmonella *mycotic aneurysms requires a combination of prolonged targeted antimicrobial therapy and definitive surgical intervention [5,7]. Medical therapy alone is associated with unacceptably high mortality due to the risk of rupture. In this case, the patient ultimately required emergent ligation of the infrarenal aorta with extra-anatomic axillo-bifemoral bypass grafting, an approach favored in contaminated fields to minimize prosthetic graft infection. The choice of antimicrobial therapy must be guided by culture and susceptibility data, with prolonged treatment typically recommended for at least 6-12 weeks [8]. The development of secondary intra-abdominal infection with ESBL-producing *Escherichia coli *further complicated management, necessitating escalation to carbapenem therapy. This highlights the dynamic nature of antimicrobial decision-making in critically ill surgical patients and the importance of close collaboration with infectious disease specialists. 

The extensive gastrointestinal complications observed - sigmoid colon ischemia, necrosis, stump insufficiency, and rectal ampullary necrosis - reflect the profound systemic and regional effects of infected aortic pathology and major vascular surgery. Compromised mesenteric perfusion, septic shock, and prolonged hypotension likely contributed to bowel ischemia. These complications significantly increased morbidity, necessitating multiple surgical interventions and prolonged intensive care support. 

This case has important occupational health implications. Accidental needle-stick injuries in agricultural settings are often underreported and underestimated in terms of infectious risk [10,11]. The reuse of needles during livestock vaccination represents a significant hazard, with the potential for direct transmission of zoonotic pathogens. Increased awareness, education, and adherence to strict safety protocols are essential to prevent similar catastrophic infections. 

## Conclusions

This case highlights a rare and devastating presentation of *Salmonella *infection resulting from accidental parenteral inoculation in an agricultural worker. The clinical course underscores the aggressive nature of *Salmonella*-associated endovascular infections and the limitations of aneurysm size criteria in the context of infection. Early recognition of unusual transmission routes, prompt identification of endovascular involvement in patients with persistent *Salmonella *bacteremia, and aggressive combined surgical and antimicrobial management are critical for survival. Furthermore, it emphasizes the importance of occupational safety measures in agricultural environments to prevent rare but life-threatening zoonotic infections. Heightened clinical vigilance and interdisciplinary collaboration remain essential in managing such complex and high-risk cases.
